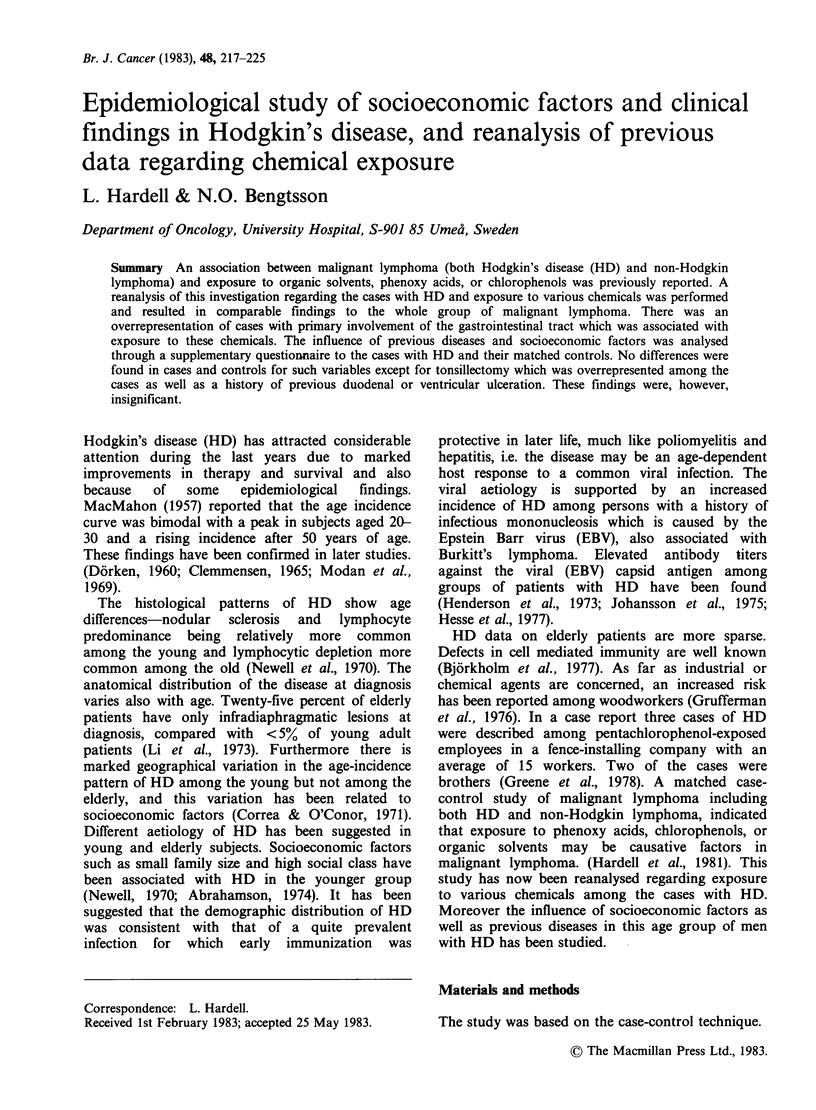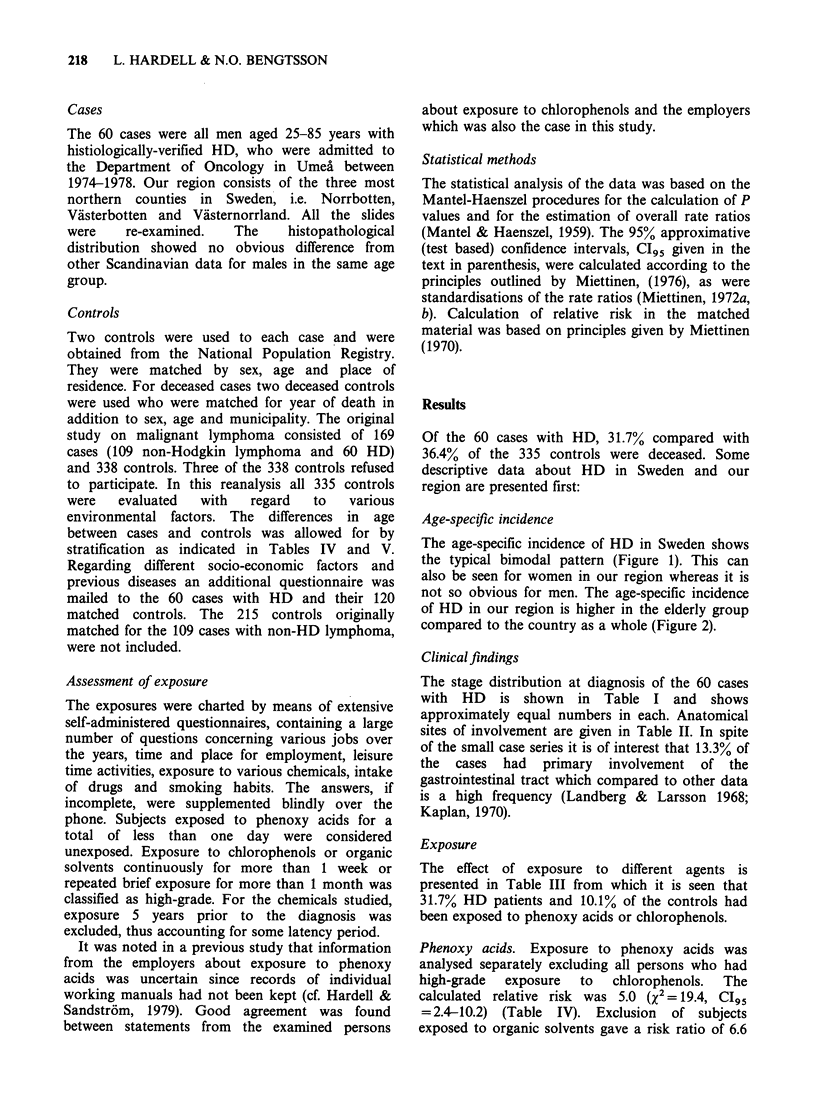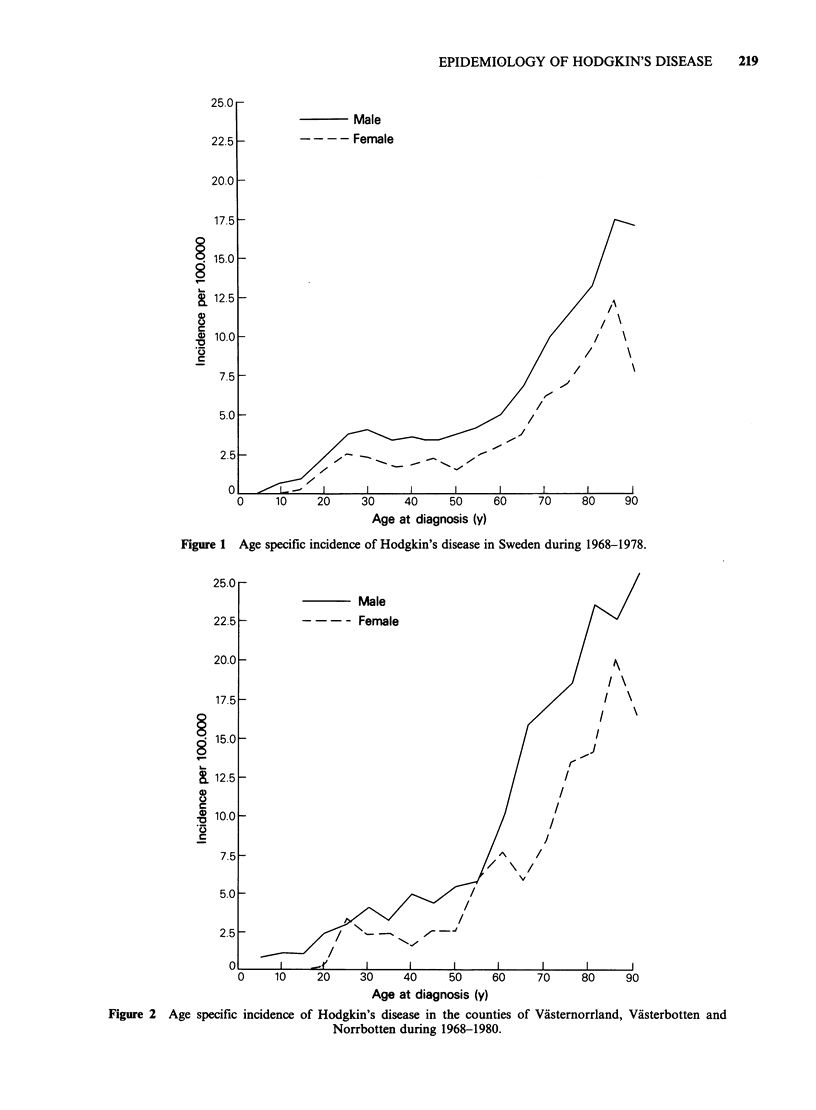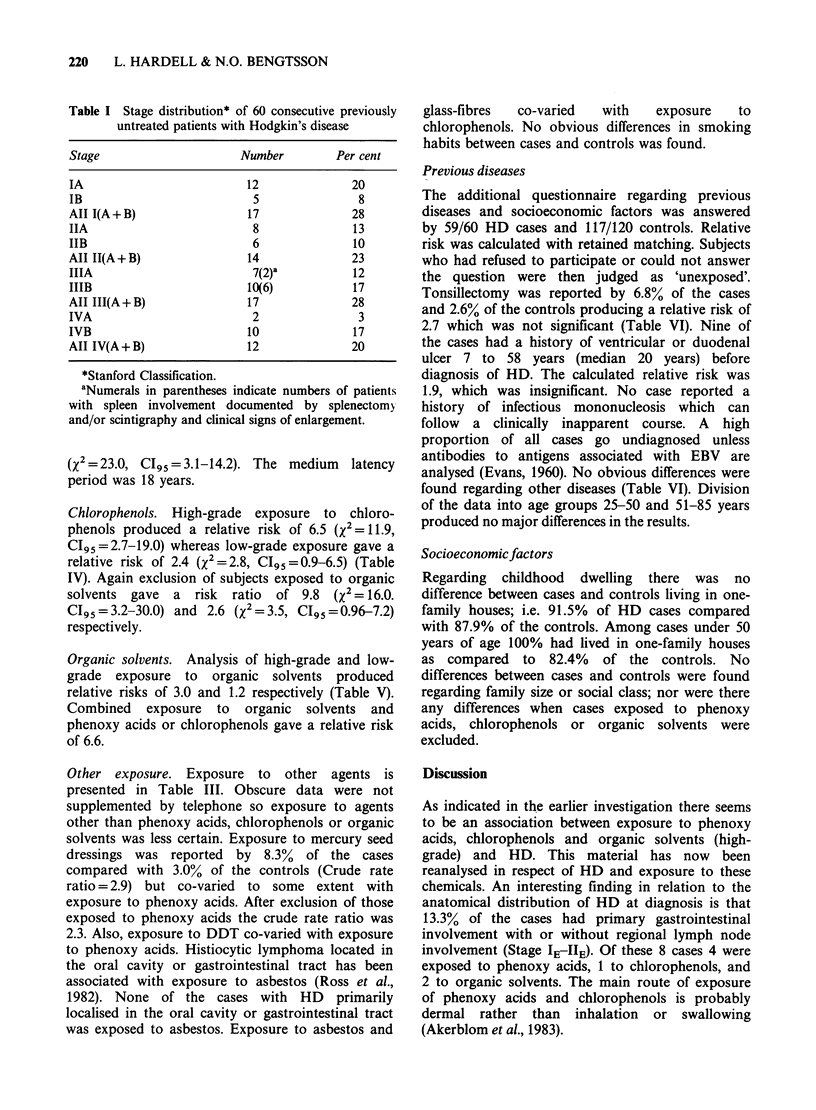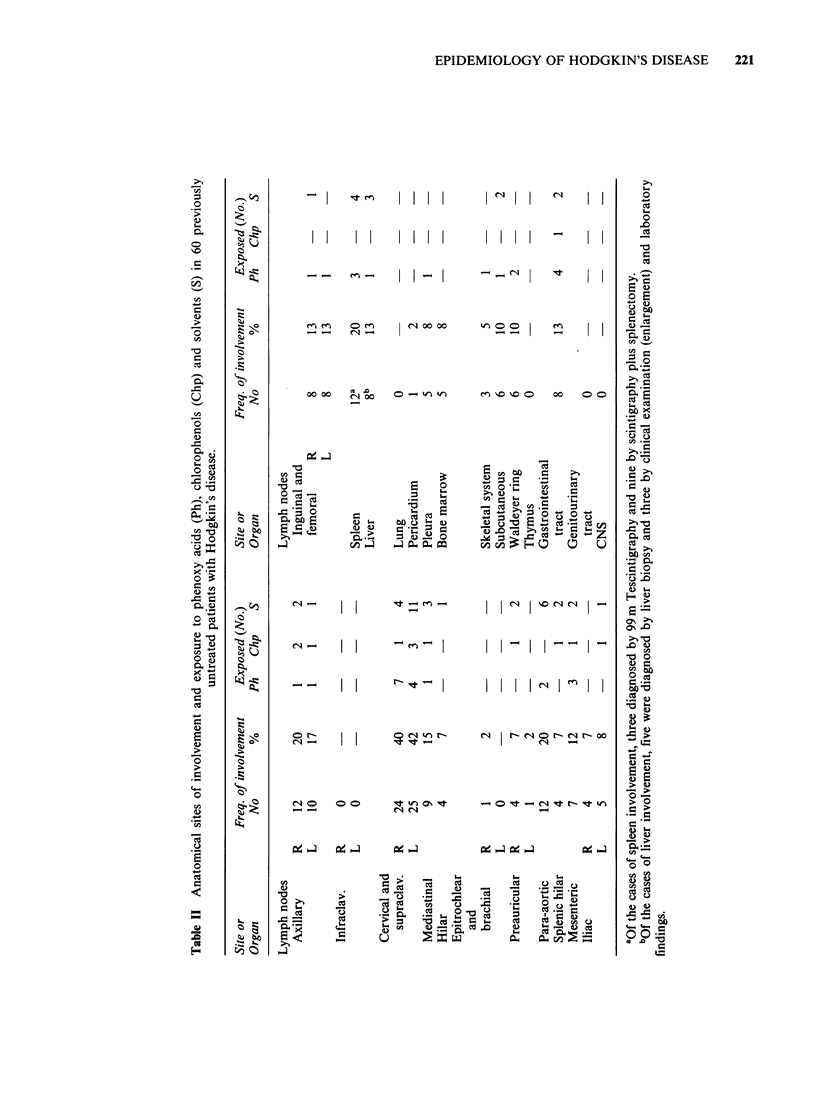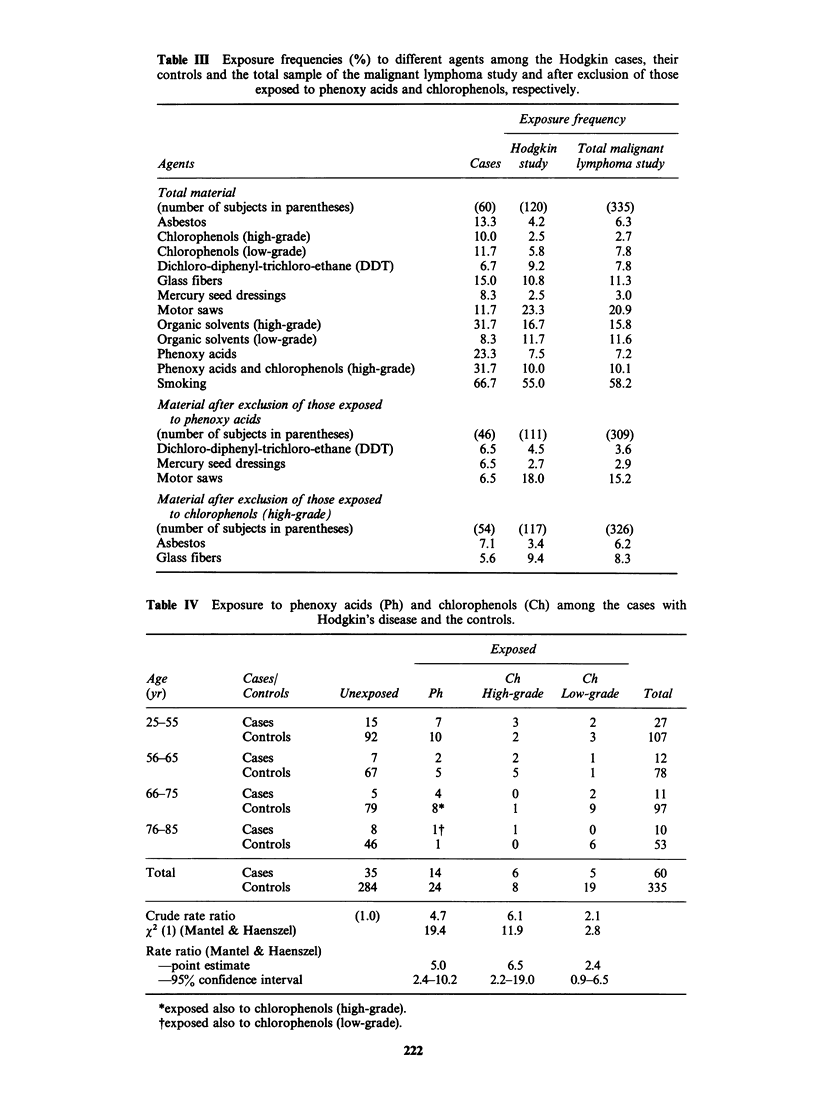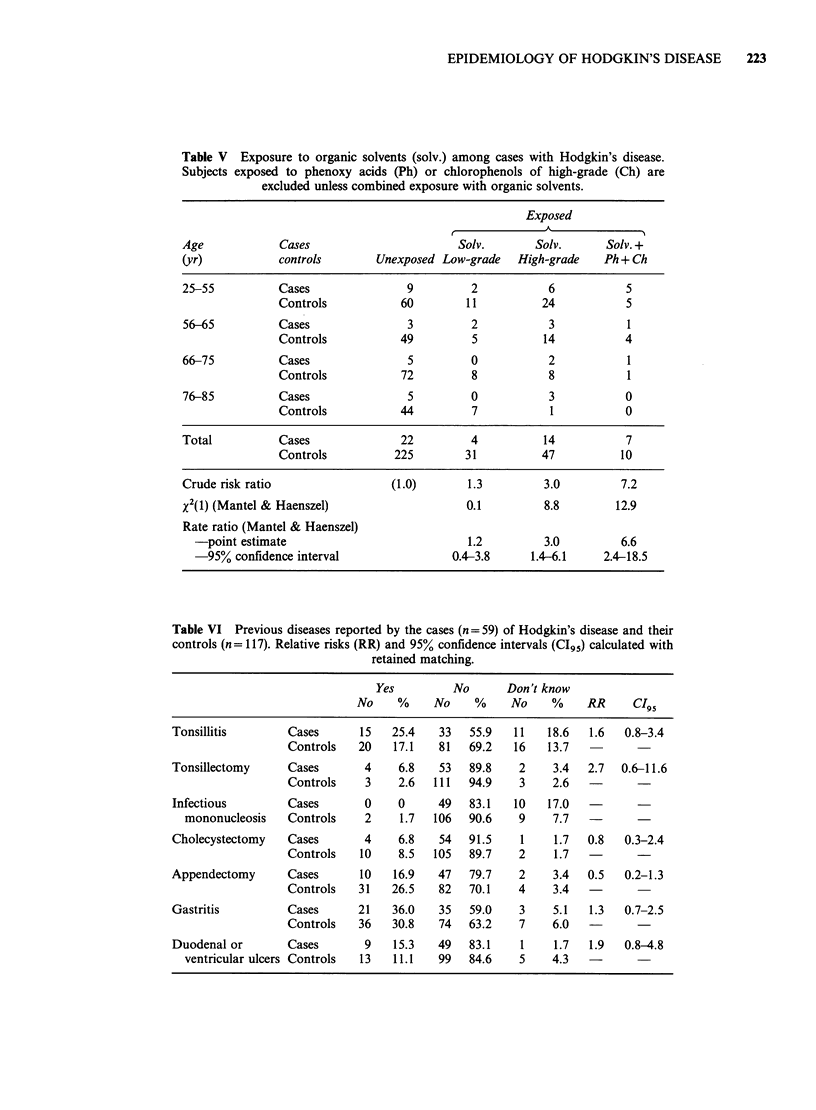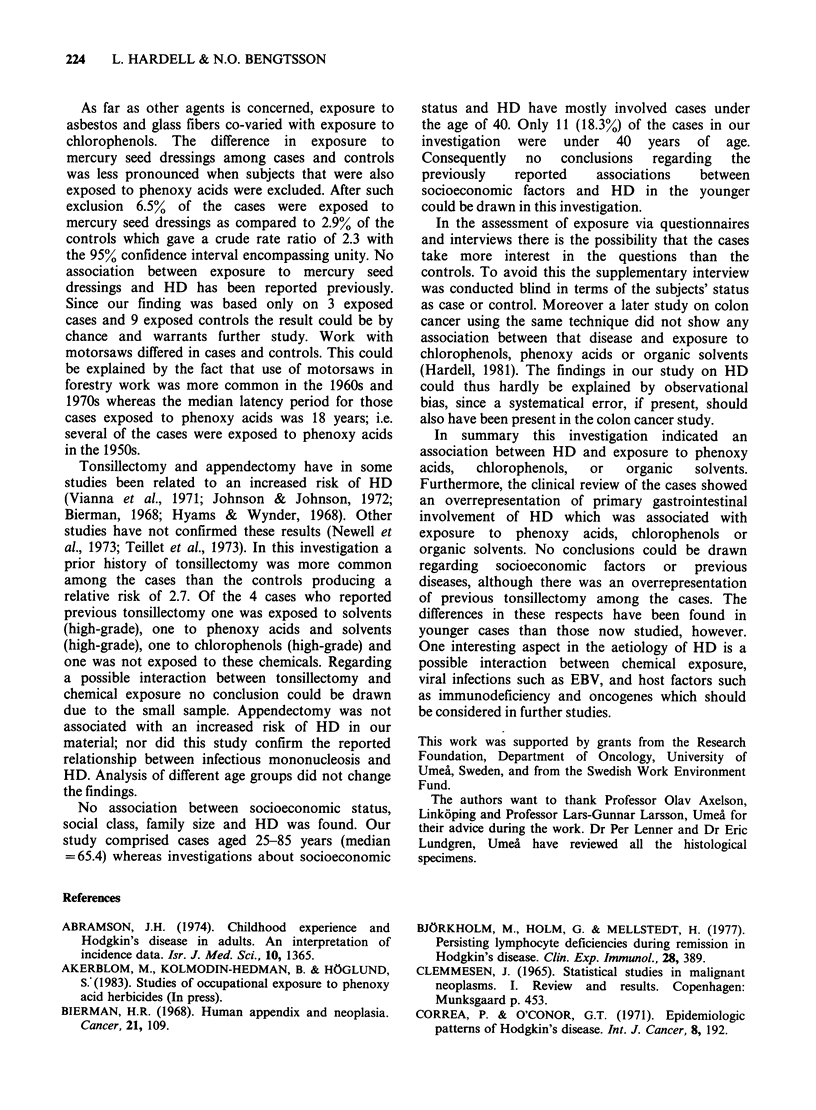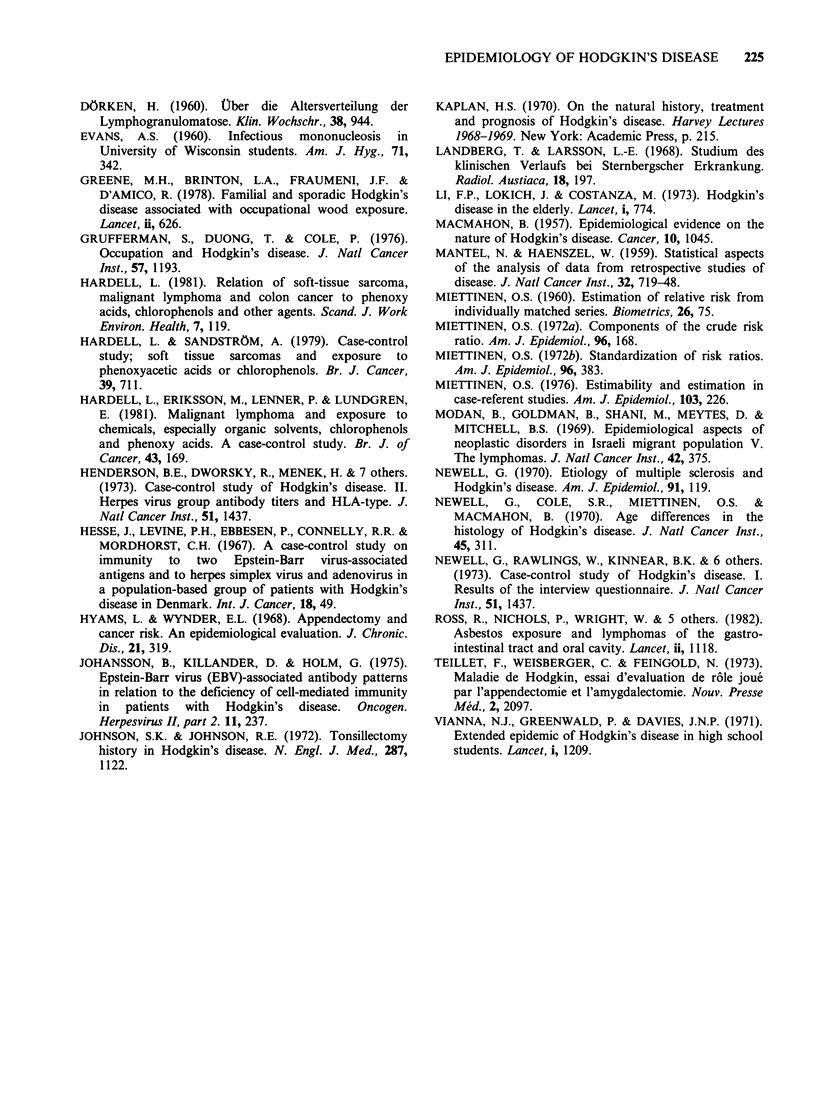# Epidemiological study of socioeconomic factors and clinical findings in Hodgkin's disease, and reanalysis of previous data regarding chemical exposure.

**DOI:** 10.1038/bjc.1983.177

**Published:** 1983-08

**Authors:** L. Hardell, N. O. Bengtsson

## Abstract

An association between malignant lymphoma (both Hodgkin's disease (HD) and non-Hodgkin lymphoma) and exposure to organic solvents, phenoxy acids, or chlorophenols was previously reported. A reanalysis of this investigation regarding the cases with HD and exposure to various chemicals was performed and resulted in comparable findings to the whole group of malignant lymphoma. There was an overrepresentation of cases with primary involvement of the gastrointestinal tract which was associated with exposure to these chemicals. The influence of previous diseases and socioeconomic factors was analysed through a supplementary questionnaire to the cases with HD and their matched controls. No differences were found in cases and controls for such variables except for tonsillectomy which was overrepresented among the cases as well as a history of previous duodenal or ventricular ulceration. These findings were, however, insignificant.


*Br. J. Cancer* (1983), **48**, 217–225

### Epidemiological study of socioeconomic factors and clinical findings in Hodgkin's disease, and reanalysis of previous data regarding chemical exposure

L. Hardell & N.O. Bengtsson

*Department of Oncology, University Hospital, S-901 85 Umeå, Sweden*

**Summary** An association between malignant lymphoma (both Hodgkin's disease (HD) and non-Hodgkin lymphoma) and exposure to organic solvents, phenoxy acids, or chlorophenols was previously reported. A reanalysis of this investigation regarding the cases with HD and exposure to various chemicals was performed and resulted in comparable findings to the whole group of malignant lymphoma. There was an overrepresentation of cases with primary involvement of the gastrointestinal tract which was associated with exposure to these chemicals. The influence of previous diseases and socioeconomic factors was analysed through a supplementary questionnaire to the cases with HD and their matched controls. No differences were found in cases and controls for such variables except for tonsillectomy which was overrepresented among the cases as well as a history of previous duodenal or ventricular ulceration. These findings were, however, insignificant.

Hodgkin's disease (HD) has attracted considerable attention during the last years due to marked improvements in therapy and survival and also because of some epidemiological findings. MacMahon (1957) reported that the age incidence curve was bimodal with a peak in subjects aged 20–30 and a rising incidence after 50 years of age. These findings have been confirmed in later studies. (Dörken, 1960; Clemmensen, 1965; Modan *et al.*, 1969).

The histological patterns of HD show age differences—nodular sclerosis and lymphocyte predominance being relatively more common among the young and lymphocytic depletion more common among the old (Newell *et al.*, 1970). The anatomical distribution of the disease at diagnosis varies also with age. Twenty-five percent of elderly patients have only infradiaphragmatic lesions at diagnosis, compared with <5% of young adult patients (Li *et al.*, 1973). Furthermore there is marked geographical variation in the age-incidence pattern of HD among the young but not among the elderly, and this variation has been related to socioeconomic factors (Correa & O'Conor, 1971). Different aetiology of HD has been suggested in young and elderly subjects. Socioeconomic factors such as small family size and high social class have been associated with HD in the younger group (Newell, 1970; Abrahamson, 1974). It has been suggested that the demographic distribution of HD was consistent with that of a quite prevalent infection for which early immunization was protective in later life, much like poliomyelitis and hepatitis, i.e. the disease may be an age-dependent host response to a common viral infection. The viral aetiology is supported by an increased incidence of HD among persons with a history of infectious mononucleosis which is caused by the Epstein Barr virus (EBV), also associated with Burkitt's lymphoma. Elevated antibody titers against the viral (EBV) capsid antigen among groups of patients with HD have been found (Henderson *et al.*, 1973; Johansson *et al.*, 1975; Hesse *et al.*, 1977).

HD data on elderly patients are more sparse. Defects in cell mediated immunity are well known (Björkholm *et al.*, 1977). As far as industrial or chemical agents are concerned, an increased risk has been reported among woodworkers (Grufferman *et al.*, 1976). In a case report three cases of HD were described among pentachlorophenol-exposed employees in a fence-installing company with an average of 15 workers. Two of the cases were brothers (Greene *et al.*, 1978). A matched case-control study of malignant lymphoma including both HD and non-Hodgkin lymphoma, indicated that exposure to phenoxy acids, chlorophenols, or organic solvents may be causative factors in malignant lymphoma. (Hardell *et al.*, 1981). This study has now been reanalysed regarding exposure to various chemicals among the cases with HD. Moreover the influence of socioeconomic factors as well as previous diseases in this age group of men with HD has been studied.

### Materials and methods

The study was based on the case-control technique.

Correspondence: L. Hardell.
Received 1st February 1983; accepted 25 May 1983.

© The Macmillan Press Ltd., 1983.

*Cases*

The 60 cases were all men aged 25–85 years with histiologically-verified HD, who were admitted to the Department of Oncology in Umeå between 1974–1978. Our region consists of the three most northern counties in Sweden, i.e. Norrbotten, Västerbotten and Västernorrland. All the slides were re-examined. The histopathological distribution showed no obvious difference from other Scandinavian data for males in the same age group.

*Controls*

Two controls were used to each case and were obtained from the National Population Registry. They were matched by sex, age and place of residence. For deceased cases two deceased controls were used who were matched for year of death in addition to sex, age and municipality. The original study on malignant lymphoma consisted of 169 cases (109 non-Hodgkin lymphoma and 60 HD) and 338 controls. Three of the 338 controls refused to participate. In this reanalysis all 335 controls were evaluated with regard to various environmental factors. The differences in age between cases and controls was allowed for by stratification as indicated in Tables IV and V. Regarding different socio-economic factors and previous diseases an additional questionnaire was mailed to the 60 cases with HD and their 120 matched controls. The 215 controls originally matched for the 109 cases with non-HD lymphoma, were not included.

*Assessment of exposure*

The exposures were charted by means of extensive self-administered questionnaires, containing a large number of questions concerning various jobs over the years, time and place for employment, leisure time activities, exposure to various chemicals, intake of drugs and smoking habits. The answers, if incomplete, were supplemented blindly over the phone. Subjects exposed to phenoxy acids for a total of less than one day were considered unexposed. Exposure to chlorophenols or organic solvents continuously for more than 1 week or repeated brief exposure for more than 1 month was classified as high-grade. For the chemicals studied, exposure 5 years prior to the diagnosis was excluded, thus accounting for some latency period.

It was noted in a previous study that information from the employers about exposure to phenoxy acids was uncertain since records of individual working manuals had not been kept (cf. Hardell & Sandström, 1979). Good agreement was found between statements from the examined persons about exposure to chlorophenols and the employers which was also the case in this study.

*Statistical methods*

The statistical analysis of the data was based on the Mantel-Haenszel procedures for the calculation of *P* values and for the estimation of overall rate ratios (Mantel & Haenszel, 1959). The 95% approximative (test based) confidence intervals, $CI_{95}$ given in the text in parenthesis, were calculated according to the principles outlined by Miettinen, (1976), as were standardisations of the rate ratios (Miettinen, 1972*a*, *b*). Calculation of relative risk in the matched material was based on principles given by Miettinen (1970).

### Results

Of the 60 cases with HD, 31.7% compared with 36.4% of the 335 controls were deceased. Some descriptive data about HD in Sweden and our region are presented first:

*Age-specific incidence*

The age-specific incidence of HD in Sweden shows the typical bimodal pattern (Figure 1). This can also be seen for women in our region whereas it is not so obvious for men. The age-specific incidence of HD in our region is higher in the elderly group compared to the country as a whole (Figure 2).

*Clinical findings*

The stage distribution at diagnosis of the 60 cases with HD is shown in Table I and shows approximately equal numbers in each. Anatomical sites of involvement are given in Table II. In spite of the small case series it is of interest that 13.3% of the cases had primary involvement of the gastrointestinal tract which compared to other data is a high frequency (Landberg & Larsson 1968; Kaplan, 1970).

*Exposure*

The effect of exposure to different agents is presented in Table III from which it is seen that 31.7% HD patients and 10.1% of the controls had been exposed to phenoxy acids or chlorophenols.

*Phenoxy acids.* Exposure to phenoxy acids was analysed separately excluding all persons who had high-grade exposure to chlorophenols. The calculated relative risk was 5.0 ($\chi^2 = 19.4$, $CI_{95} = 2.4$–$10.2$) (Table IV). Exclusion of subjects exposed to organic solvents gave a risk ratio of 6.6

**Figure 1** Age specific incidence of Hodgkin's disease in Sweden during 1968–1978.

**Figure 2** Age specific incidence of Hodgkin's disease in the counties of Västernorrland, Västerbotten and Norrbotten during 1968–1980.

**Table I** Stage distribution* of 60 consecutive previously untreated patients with Hodgkin's disease

| *Stage* | *Number* | *Per cent* |
|---|---|---|
| IA | 12 | 20 |
| IB | 5 | 8 |
| All I(A+B) | 17 | 28 |
| IIA | 8 | 13 |
| IIB | 6 | 10 |
| All II(A+B) | 14 | 23 |
| IIIA | 7(2)[a] | 12 |
| IIIB | 10(6) | 17 |
| All III(A+B) | 17 | 28 |
| IVA | 2 | 3 |
| IVB | 10 | 17 |
| All IV(A+B) | 12 | 20 |

*Stanford Classification.
[a]Numerals in parentheses indicate numbers of patients with spleen involvement documented by splenectomy and/or scintigraphy and clinical signs of enlargement.

($\chi^2=23.0$, $CI_{95}=3.1$–$14.2$). The medium latency period was 18 years.

*Chlorophenols.* High-grade exposure to chlorophenols produced a relative risk of 6.5 ($\chi^2=11.9$, $CI_{95}=2.7$–$19.0$) whereas low-grade exposure gave a relative risk of 2.4 ($\chi^2=2.8$, $CI_{95}=0.9$–$6.5$) (Table IV). Again exclusion of subjects exposed to organic solvents gave a risk ratio of 9.8 ($\chi^2=16.0$. $CI_{95}=3.2$–$30.0$) and 2.6 ($\chi^2=3.5$, $CI_{95}=0.96$–$7.2$) respectively.

*Organic solvents.* Analysis of high-grade and low-grade exposure to organic solvents produced relative risks of 3.0 and 1.2 respectively (Table V). Combined exposure to organic solvents and phenoxy acids or chlorophenols gave a relative risk of 6.6.

*Other exposure.* Exposure to other agents is presented in Table III. Obscure data were not supplemented by telephone so exposure to agents other than phenoxy acids, chlorophenols or organic solvents was less certain. Exposure to mercury seed dressings was reported by 8.3% of the cases compared with 3.0% of the controls (Crude rate ratio = 2.9) but co-varied to some extent with exposure to phenoxy acids. After exclusion of those exposed to phenoxy acids the crude rate ratio was 2.3. Also, exposure to DDT co-varied with exposure to phenoxy acids. Histiocytic lymphoma located in the oral cavity or gastrointestinal tract has been associated with exposure to asbestos (Ross *et al.*, 1982). None of the cases with HD primarily localised in the oral cavity or gastrointestinal tract was exposed to asbestos. Exposure to asbestos and glass-fibres co-varied with exposure to chlorophenols. No obvious differences in smoking habits between cases and controls was found.

*Previous diseases*

The additional questionnaire regarding previous diseases and socioeconomic factors was answered by 59/60 HD cases and 117/120 controls. Relative risk was calculated with retained matching. Subjects who had refused to participate or could not answer the question were then judged as 'unexposed'. Tonsillectomy was reported by 6.8% of the cases and 2.6% of the controls producing a relative risk of 2.7 which was not significant (Table VI). Nine of the cases had a history of ventricular or duodenal ulcer 7 to 58 years (median 20 years) before diagnosis of HD. The calculated relative risk was 1.9, which was insignificant. No case reported a history of infectious mononucleosis which can follow a clinically inapparent course. A high proportion of all cases go undiagnosed unless antibodies to antigens associated with EBV are analysed (Evans, 1960). No obvious differences were found regarding other diseases (Table VI). Division of the data into age groups 25–50 and 51–85 years produced no major differences in the results.

*Socioeconomic factors*

Regarding childhood dwelling there was no difference between cases and controls living in one-family houses; i.e. 91.5% of HD cases compared with 87.9% of the controls. Among cases under 50 years of age 100% had lived in one-family houses as compared to 82.4% of the controls. No differences between cases and controls were found regarding family size or social class; nor were there any differences when cases exposed to phenoxy acids, chlorophenols or organic solvents were excluded.

### Discussion

As indicated in the earlier investigation there seems to be an association between exposure to phenoxy acids, chlorophenols and organic solvents (high-grade) and HD. This material has now been reanalysed in respect of HD and exposure to these chemicals. An interesting finding in relation to the anatomical distribution of HD at diagnosis is that 13.3% of the cases had primary gastrointestinal involvement with or without regional lymph node involvement (Stage $I_E$–$II_E$). Of these 8 cases 4 were exposed to phenoxy acids, 1 to chlorophenols, and 2 to organic solvents. The main route of exposure of phenoxy acids and chlorophenols is probably dermal rather than inhalation or swallowing (Akerblom *et al.*, 1983).

**Table II** Anatomical sites of involvement and exposure to phenoxy acids (Ph), chlorophenols (Chp) and solvents (S) in 60 previously untreated patients with Hodgkin's disease.

| *Site or Organ* | | *Freq. of involvement* No | *Freq. of involvement* % | *Exposed (No.)* Ph | *Exposed (No.)* Chp | *Exposed (No.)* S | *Site or Organ* | | *Freq. of involvement* No | *Freq. of involvement* % | *Exposed (No.)* Ph | *Exposed (No.)* Chp | *Exposed (No.)* S |
|---|---|---|---|---|---|---|---|---|---|---|---|---|---|
| Lymph nodes | | | | | | | Lymph nodes | | | | | | |
| Axillary | R | 12 | 20 | 1 | 2 | 2 | Inguinal and | | | | | | |
| | L | 10 | 17 | 1 | 1 | 1 | femoral | R | 8 | 13 | 1 | — | 1 |
| | | | | | | | | L | 8 | 13 | 1 | — | — |
| Infraclav. | R | 0 | — | — | — | — | | | | | | | |
| | L | 0 | — | — | — | — | Spleen | | 12[a] | 20 | 3 | — | 4 |
| | | | | | | | Liver | | 8[b] | 13 | 1 | — | 3 |
| Cervical and | | | | | | | | | | | | | |
| supraclav. | R | 24 | 40 | 7 | 1 | 4 | Lung | | 0 | — | — | — | — |
| | L | 25 | 42 | 4 | 3 | 11 | Pericardium | | 1 | 2 | — | — | — |
| Mediastinal | | 9 | 15 | 1 | 1 | 3 | Pleura | | 5 | 8 | 1 | — | — |
| Hilar | | 4 | 7 | — | — | 1 | Bone marrow | | 5 | 8 | — | — | — |
| Epitrochlear and | | | | | | | | | | | | | |
| brachial | R | 1 | 2 | — | — | — | Skeletal system | | 3 | 5 | 1 | — | — |
| | L | 0 | — | — | — | — | Subcutaneous | | 6 | 10 | 1 | — | 2 |
| Preauricular | R | 4 | 7 | — | 1 | 2 | Waldeyer ring | | 6 | 10 | 2 | — | — |
| | L | 1 | 2 | — | — | — | Thymus | | 0 | — | — | — | — |
| Para-aortic | | 12 | 20 | 2 | — | 6 | Gastrointestinal | | | | | | |
| Splenic hilar | | 4 | 7 | — | 1 | 2 | tract | | 8 | 13 | 4 | 1 | 2 |
| Mesenteric | | 7 | 12 | 3 | 1 | 2 | Genitourinary | | | | | | |
| Iliac | R | 4 | 7 | — | — | — | tract | | 0 | — | — | — | — |
| | L | 5 | 8 | — | 1 | 1 | CNS | | 0 | — | — | — | — |

[a]Of the cases of spleen involvement, three diagnosed by 99 m Tescintigraphy and nine by scintigraphy plus splenectomy.

[b]Of the cases of liver involvement, five were diagnosed by liver biopsy and three by clinical examination (enlargement) and laboratory findings.

**Table III** Exposure frequencies (%) to different agents among the Hodgkin cases, their controls and the total sample of the malignant lymphoma study and after exclusion of those exposed to phenoxy acids and chlorophenols, respectively.

| | | *Exposure frequency* | |
|---|---|---|---|
| *Agents* | *Cases* | *Hodgkin study* | *Total malignant lymphoma study* |
| *Total material* | | | |
| (number of subjects in parentheses) | (60) | (120) | (335) |
| Asbestos | 13.3 | 4.2 | 6.3 |
| Chlorophenols (high-grade) | 10.0 | 2.5 | 2.7 |
| Chlorophenols (low-grade) | 11.7 | 5.8 | 7.8 |
| Dichloro-diphenyl-trichloro-ethane (DDT) | 6.7 | 9.2 | 7.8 |
| Glass fibers | 15.0 | 10.8 | 11.3 |
| Mercury seed dressings | 8.3 | 2.5 | 3.0 |
| Motor saws | 11.7 | 23.3 | 20.9 |
| Organic solvents (high-grade) | 31.7 | 16.7 | 15.8 |
| Organic solvents (low-grade) | 8.3 | 11.7 | 11.6 |
| Phenoxy acids | 23.3 | 7.5 | 7.2 |
| Phenoxy acids and chlorophenols (high-grade) | 31.7 | 10.0 | 10.1 |
| Smoking | 66.7 | 55.0 | 58.2 |
| *Material after exclusion of those exposed to phenoxy acids* | | | |
| (number of subjects in parentheses) | (46) | (111) | (309) |
| Dichloro-diphenyl-trichloro-ethane (DDT) | 6.5 | 4.5 | 3.6 |
| Mercury seed dressings | 6.5 | 2.7 | 2.9 |
| Motor saws | 6.5 | 18.0 | 15.2 |
| *Material after exclusion of those exposed to chlorophenols (high-grade)* | | | |
| (number of subjects in parentheses) | (54) | (117) | (326) |
| Asbestos | 7.1 | 3.4 | 6.2 |
| Glass fibers | 5.6 | 9.4 | 8.3 |

**Table IV** Exposure to phenoxy acids (Ph) and chlorophenols (Ch) among the cases with Hodgkin's disease and the controls.

| | | | *Exposed* | | | |
|---|---|---|---|---|---|---|
| *Age (yr)* | *Cases/ Controls* | *Unexposed* | *Ph* | *Ch High-grade* | *Ch Low-grade* | *Total* |
| 25–55 | Cases | 15 | 7 | 3 | 2 | 27 |
| | Controls | 92 | 10 | 2 | 3 | 107 |
| 56–65 | Cases | 7 | 2 | 2 | 1 | 12 |
| | Controls | 67 | 5 | 5 | 1 | 78 |
| 66–75 | Cases | 5 | 4 | 0 | 2 | 11 |
| | Controls | 79 | 8* | 1 | 9 | 97 |
| 76–85 | Cases | 8 | 1† | 1 | 0 | 10 |
| | Controls | 46 | 1 | 0 | 6 | 53 |
| Total | Cases | 35 | 14 | 6 | 5 | 60 |
| | Controls | 284 | 24 | 8 | 19 | 335 |
| Crude rate ratio | | (1.0) | 4.7 | 6.1 | 2.1 | |
| $\chi^2$ (1) (Mantel & Haenszel) | | | 19.4 | 11.9 | 2.8 | |
| Rate ratio (Mantel & Haenszel) | | | | | | |
| —point estimate | | | 5.0 | 6.5 | 2.4 | |
| —95% confidence interval | | | 2.4–10.2 | 2.2–19.0 | 0.9–6.5 | |

*exposed also to chlorophenols (high-grade).
†exposed also to chlorophenols (low-grade).

**Table V** Exposure to organic solvents (solv.) among cases with Hodgkin's disease. Subjects exposed to phenoxy acids (Ph) or chlorophenols of high-grade (Ch) are excluded unless combined exposure with organic solvents.

| *Age (yr)* | *Cases controls* | *Unexposed* | *Exposed* *Solv. Low-grade* | *Solv. High-grade* | *Solv.+ Ph+Ch* |
|---|---|---|---|---|---|
| 25–55 | Cases | 9 | 2 | 6 | 5 |
| | Controls | 60 | 11 | 24 | 5 |
| 56–65 | Cases | 3 | 2 | 3 | 1 |
| | Controls | 49 | 5 | 14 | 4 |
| 66–75 | Cases | 5 | 0 | 2 | 1 |
| | Controls | 72 | 8 | 8 | 1 |
| 76–85 | Cases | 5 | 0 | 3 | 0 |
| | Controls | 44 | 7 | 1 | 0 |
| Total | Cases | 22 | 4 | 14 | 7 |
| | Controls | 225 | 31 | 47 | 10 |
| Crude risk ratio | | (1.0) | 1.3 | 3.0 | 7.2 |
| $\chi^2(1)$ (Mantel & Haenszel) | | | 0.1 | 8.8 | 12.9 |
| Rate ratio (Mantel & Haenszel) | | | | | |
| —point estimate | | | 1.2 | 3.0 | 6.6 |
| —95% confidence interval | | | 0.4–3.8 | 1.4–6.1 | 2.4–18.5 |

**Table VI** Previous diseases reported by the cases ($n=59$) of Hodgkin's disease and their controls ($n=117$). Relative risks (RR) and 95% confidence intervals ($CI_{95}$) calculated with retained matching.

| | | *Yes* | | *No* | | *Don't know* | | | |
|---|---|---|---|---|---|---|---|---|---|
| | | *No* | % | *No* | % | *No* | % | *RR* | $CI_{95}$ |
| Tonsillitis | Cases | 15 | 25.4 | 33 | 55.9 | 11 | 18.6 | 1.6 | 0.8–3.4 |
| | Controls | 20 | 17.1 | 81 | 69.2 | 16 | 13.7 | — | — |
| Tonsillectomy | Cases | 4 | 6.8 | 53 | 89.8 | 2 | 3.4 | 2.7 | 0.6–11.6 |
| | Controls | 3 | 2.6 | 111 | 94.9 | 3 | 2.6 | — | — |
| Infectious mononucleosis | Cases | 0 | 0 | 49 | 83.1 | 10 | 17.0 | — | — |
| | Controls | 2 | 1.7 | 106 | 90.6 | 9 | 7.7 | — | — |
| Cholecystectomy | Cases | 4 | 6.8 | 54 | 91.5 | 1 | 1.7 | 0.8 | 0.3–2.4 |
| | Controls | 10 | 8.5 | 105 | 89.7 | 2 | 1.7 | — | — |
| Appendectomy | Cases | 10 | 16.9 | 47 | 79.7 | 2 | 3.4 | 0.5 | 0.2–1.3 |
| | Controls | 31 | 26.5 | 82 | 70.1 | 4 | 3.4 | — | — |
| Gastritis | Cases | 21 | 36.0 | 35 | 59.0 | 3 | 5.1 | 1.3 | 0.7–2.5 |
| | Controls | 36 | 30.8 | 74 | 63.2 | 7 | 6.0 | — | — |
| Duodenal or ventricular ulcers | Cases | 9 | 15.3 | 49 | 83.1 | 1 | 1.7 | 1.9 | 0.8–4.8 |
| | Controls | 13 | 11.1 | 99 | 84.6 | 5 | 4.3 | — | — |

As far as other agents is concerned, exposure to asbestos and glass fibers co-varied with exposure to chlorophenols. The difference in exposure to mercury seed dressings among cases and controls was less pronounced when subjects that were also exposed to phenoxy acids were excluded. After such exclusion 6.5% of the cases were exposed to mercury seed dressings as compared to 2.9% of the controls which gave a crude rate ratio of 2.3 with the 95% confidence interval encompassing unity. No association between exposure to mercury seed dressings and HD has been reported previously. Since our finding was based only on 3 exposed cases and 9 exposed controls the result could be by chance and warrants further study. Work with motorsaws differed in cases and controls. This could be explained by the fact that use of motorsaws in forestry work was more common in the 1960s and 1970s whereas the median latency period for those cases exposed to phenoxy acids was 18 years; i.e. several of the cases were exposed to phenoxy acids in the 1950s.

Tonsillectomy and appendectomy have in some studies been related to an increased risk of HD (Vianna *et al.*, 1971; Johnson & Johnson, 1972; Bierman, 1968; Hyams & Wynder, 1968). Other studies have not confirmed these results (Newell *et al.*, 1973; Teillet *et al.*, 1973). In this investigation a prior history of tonsillectomy was more common among the cases than the controls producing a relative risk of 2.7. Of the 4 cases who reported previous tonsillectomy one was exposed to solvents (high-grade), one to phenoxy acids and solvents (high-grade), one to chlorophenols (high-grade) and one was not exposed to these chemicals. Regarding a possible interaction between tonsillectomy and chemical exposure no conclusion could be drawn due to the small sample. Appendectomy was not associated with an increased risk of HD in our material; nor did this study confirm the reported relationship between infectious mononucleosis and HD. Analysis of different age groups did not change the findings.

No association between socioeconomic status, social class, family size and HD was found. Our study comprised cases aged 25–85 years (median = 65.4) whereas investigations about socioeconomic status and HD have mostly involved cases under the age of 40. Only 11 (18.3%) of the cases in our investigation were under 40 years of age. Consequently no conclusions regarding the previously reported associations between socioeconomic factors and HD in the younger could be drawn in this investigation.

In the assessment of exposure via questionnaires and interviews there is the possibility that the cases take more interest in the questions than the controls. To avoid this the supplementary interview was conducted blind in terms of the subjects' status as case or control. Moreover a later study on colon cancer using the same technique did not show any association between that disease and exposure to chlorophenols, phenoxy acids or organic solvents (Hardell, 1981). The findings in our study on HD could thus hardly be explained by observational bias, since a systematical error, if present, should also have been present in the colon cancer study.

In summary this investigation indicated an association between HD and exposure to phenoxy acids, chlorophenols, or organic solvents. Furthermore, the clinical review of the cases showed an overrepresentation of primary gastrointestinal involvement of HD which was associated with exposure to phenoxy acids, chlorophenols or organic solvents. No conclusions could be drawn regarding socioeconomic factors or previous diseases, although there was an overrepresentation of previous tonsillectomy among the cases. The differences in these respects have been found in younger cases than those now studied, however. One interesting aspect in the aetiology of HD is a possible interaction between chemical exposure, viral infections such as EBV, and host factors such as immunodeficiency and oncogenes which should be considered in further studies.

This work was supported by grants from the Research Foundation, Department of Oncology, University of Umeå, Sweden, and from the Swedish Work Environment Fund.

The authors want to thank Professor Olav Axelson, Linköping and Professor Lars-Gunnar Larsson, Umeå for their advice during the work. Dr Per Lenner and Dr Eric Lundgren, Umeå have reviewed all the histological specimens.

### References

ABRAMSON, J.H. (1974). Childhood experience and Hodgkin's disease in adults. An interpretation of incidence data. *Isr. J. Med. Sci.*, **10**, 1365.

AKERBLOM, M., KOLMODIN-HEDMAN, B. & HÖGLUND, S. (1983). Studies of occupational exposure to phenoxy acid herbicides (In press).

BIERMAN, H.R. (1968). Human appendix and neoplasia. *Cancer*, **21**, 109.

BJÖRKHOLM, M., HOLM, G. & MELLSTEDT, H. (1977). Persisting lymphocyte deficiencies during remission in Hodgkin's disease. *Clin. Exp. Immunol.*, **28**, 389.

CLEMMESEN, J. (1965). Statistical studies in malignant neoplasms. I. Review and results. Copenhagen: Munksgaard p. 453.

CORREA, P. & O'CONOR, G.T. (1971). Epidemiologic patterns of Hodgkin's disease. *Int. J. Cancer*, **8**, 192.

DÖRKEN, H. (1960). Über die Altersverteilung der Lymphogranulomatose. *Klin. Wochschr.*, **38**, 944.

EVANS, A.S. (1960). Infectious mononucleosis in University of Wisconsin students. *Am. J. Hyg.*, **71**, 342.

GREENE, M.H., BRINTON, L.A., FRAUMENI, J.F. & D'AMICO, R. (1978). Familial and sporadic Hodgkin's disease associated with occupational wood exposure. *Lancet*, **ii**, 626.

GRUFFERMAN, S., DUONG, T. & COLE, P. (1976). Occupation and Hodgkin's disease. *J. Natl Cancer Inst.*, **57**, 1193.

HARDELL, L. (1981). Relation of soft-tissue sarcoma, malignant lymphoma and colon cancer to phenoxy acids, chlorophenols and other agents. *Scand. J. Work Environ. Health*, **7**, 119.

HARDELL, L. & SANDSTRÖM, A. (1979). Case-control study; soft tissue sarcomas and exposure to phenoxyacetic acids or chlorophenols. *Br. J. Cancer*, **39**, 711.

HARDELL, L., ERIKSSON, M., LENNER, P. & LUNDGREN, E. (1981). Malignant lymphoma and exposure to chemicals, especially organic solvents, chlorophenols and phenoxy acids. A case-control study. *Br. J. of Cancer*, **43**, 169.

HENDERSON, B.E., DWORSKY, R., MENEK, H. & 7 others. (1973). Case-control study of Hodgkin's disease. II. Herpes virus group antibody titers and HLA-type. *J. Natl Cancer Inst.*, **51**, 1437.

HESSE, J., LEVINE, P.H., EBBESEN, P., CONNELLY, R.R. & MORDHORST, C.H. (1967). A case-control study on immunity to two Epstein-Barr virus-associated antigens and to herpes simplex virus and adenovirus in a population-based group of patients with Hodgkin's disease in Denmark. *Int. J. Cancer*, **18**, 49.

HYAMS, L. & WYNDER, E.L. (1968). Appendectomy and cancer risk. An epidemiological evaluation. *J. Chronic. Dis.*, **21**, 319.

JOHANSSON, B., KILLANDER, D. & HOLM, G. (1975). Epstein-Barr virus (EBV)-associated antibody patterns in relation to the deficiency of cell-mediated immunity in patients with Hodgkin's disease. *Oncogen. Herpesvirus II, part 2*. **11**, 237.

JOHNSON, S.K. & JOHNSON, R.E. (1972). Tonsillectomy history in Hodgkin's disease. *N. Engl. J. Med.*, **287**, 1122.

KAPLAN, H.S. (1970). On the natural history, treatment and prognosis of Hodgkin's disease. *Harvey Lectures 1968–1969*. New York: Academic Press, p. 215.

LANDBERG, T. & LARSSON, L.-E. (1968). Studium des klinischen Verlaufs bei Sternbergscher Erkrankung. *Radiol. Austiaca*, **18**, 197.

LI, F.P., LOKICH, J. & COSTANZA, M. (1973). Hodgkin's disease in the elderly. *Lancet*, **i**, 774.

MACMAHON, B. (1957). Epidemiological evidence on the nature of Hodgkin's disease. *Cancer*, **10**, 1045.

MANTEL, N. & HAENSZEL, W. (1959). Statistical aspects of the analysis of data from retrospective studies of disease. *J. Natl Cancer Inst.*, **32**, 719–48.

MIETTINEN, O.S. (1960). Estimation of relative risk from individually matched series. *Biometrics*, **26**, 75.

MIETTINEN, O.S. (1972*a*). Components of the crude risk ratio. *Am. J. Epidemiol.*, **96**, 168.

MIETTINEN, O.S. (1972*b*). Standardization of risk ratios. *Am. J. Epidemiol.*, **96**, 383.

MIETTINEN, O.S. (1976). Estimability and estimation in case-referent studies. *Am. J. Epidemiol.*, **103**, 226.

MODAN, B., GOLDMAN, B., SHANI, M., MEYTES, D. & MITCHELL, B.S. (1969). Epidemiological aspects of neoplastic disorders in Israeli migrant population V. The lymphomas. *J. Natl Cancer Inst.*, **42**, 375.

NEWELL, G. (1970). Etiology of multiple sclerosis and Hodgkin's disease. *Am. J. Epidemiol.*, **91**, 119.

NEWELL, G., COLE, S.R., MIETTINEN, O.S. & MACMAHON, B. (1970). Age differences in the histology of Hodgkin's disease. *J. Natl Cancer Inst.*, **45**, 311.

NEWELL, G., RAWLINGS, W., KINNEAR, B.K. & 6 others. (1973). Case-control study of Hodgkin's disease. I. Results of the interview questionnaire. *J. Natl Cancer Inst.*, **51**, 1437.

ROSS, R., NICHOLS, P., WRIGHT, W. & 5 others. (1982). Asbestos exposure and lymphomas of the gastro-intestinal tract and oral cavity. *Lancet*, **ii**, 1118.

TEILLET, F., WEISBERGER, C. & FEINGOLD, N. (1973). Maladie de Hodgkin, essai d'evaluation de rôle joué par l'appendectomie et l'amygdalectomie. *Nouv. Presse Méd.*, **2**, 2097.

VIANNA, N.J., GREENWALD, P. & DAVIES, J.N.P. (1971). Extended epidemic of Hodgkin's disease in high school students. *Lancet*, **i**, 1209.